# Membranous nephropathy associated with multicentric Castleman’s disease that was successfully treated with tocilizumab: a case report and review of the literature

**DOI:** 10.1186/s12882-021-02423-w

**Published:** 2021-06-09

**Authors:** Ryosuke Saiki, Kan Katayama, Yosuke Hirabayashi, Keiko Oda, Mika Fujimoto, Tomohiro Murata, Ayako Nakajima, Kaoru Dohi

**Affiliations:** 1 Department of Cardiology and Nephrology, Mie University Graduate School of Medicine, 2-174 Edobashi, Tsu, Mie 514-8507 Japan; 2 Center for Rheumatic Diseases, Mie University Hospital, Tsu, Japan

**Keywords:** Castleman’s disease - membranous nephropathy, Proteinuria - tocilizumab

## Abstract

**Background:**

Multicentric Castleman’s disease is a life-threatening disorder involving a systemic inflammatory response and multiple organ failure caused by the overproduction of interleukin-6. Although renal complications of Castleman’s disease include AA amyloidosis, thrombotic microangiopathy, and membranoproliferative glomerulonephritis, membranous nephropathy is relatively rare. We experienced a case of secondary membranous nephropathy associated with Castleman’s disease.

**Case presentation:**

The patient was a 43-year-old Japanese man who had shown a high zinc sulfate value in turbidity test, polyclonal hypergammaglobulinemia, anemia, and proteinuria. A physical examination revealed diffuse lymphadenopathy, an enlarged spleen and papulae of the body trunk. A skin biopsy of a papule on the patient’s back showed plasma cells in the perivascular area and he was diagnosed with multicentric Castleman’s disease, plasma cell variant. Kidney biopsy showed the appearance of bubbling in the glomerular basement membranes in Periodic acid methenamine silver stain and electron microscopy revealed electron dense deposits within and outside the glomerular basement membranes. Since immunofluorescence study showed predominant granular deposition of IgG1 and IgG2, he was diagnosed with secondary membranous nephropathy associated with Castleman’s disease. He was initially treated with prednisolone alone, however his biochemical abnormalities did not improve. After intravenous tocilizumab (700 mg every 2 weeks) was started, his C-reactive protein elevation, anemia, and polyclonal gammopathy improved. Furthermore, his urinary protein level declined from 1.58 g/gCr to 0.13 g/gCr. The prednisolone dose was gradually tapered, then discontinued. He has been stable without a recurrence of proteinuria for more than 6 months.

**Conclusions:**

Tocilizumab might be a treatment option for secondary membranous nephropathy associated with Castleman’s disease.

## Background

Castleman’s disease (CD) consists of a lymphoproliferative disorder that was first reported by Castleman et al. [1]. CD is classified into two types: unicentric CD (UCD) and multicentric CD (MCD). While UCD is thought to be a benign disease because curative resection is possible for most patients [2], MCD is a life-threatening disorder involving a systemic inflammatory response and multiple organ failure caused by the overproduction of interleukin-6 (IL-6) [3]. Previous studies have shown that renal complications of CD mainly include AA amyloidosis, thrombotic microangiopathy (TMA), and membranoproliferative glomerulonephritis (MPGN) [4, 5]. The treatment of renal complications of CD has not been clarified. We herein report a case of secondary membranous nephropathy (MN) associated with CD that was successfully treated with tocilizumab.

## Case presentation

The patient was a 43-year-old Japanese man who had shown a high zinc sulfate value in turbidity test in a health checkup 4 years previously. He was examined by a local doctor who pointed out hypergammaglobulinemia and anemia. He had taken colchicine and febuxostat to treat gout 3 years previously. After presenting mild hematuria and proteinuria 2 years previously, he was referred to our hospital for further examination and treatment. The patient’s height was 170 cm, and his body weight was 85 kg. His blood pressure was 129/83 mmHg, his pulse was 95 beats/minute, and his body temperature was 36.2 °C. He sometimes experienced night sweat. A physical examination revealed diffuse lymphadenopathy, an enlarged spleen and papulae of the body trunk. The laboratory data are shown in Table 1. There was no monoclonal peak on immunoelectrophoresis in serum and urine tests. Renal ultrasound showed that the kidneys were of normal size, with normal renal arterial resistive indices. Computed tomography of the chest, abdomen, and pelvis showed cervical, axillary, and paraaortic lymphadenopathies and splenomegaly.

**Table 1:** Laboratory data before the kidney biopsy

Urinary examination	–	Blood chemistry	–
pH (4.5–7.5)	7	HbA1c (%, 4.9–6.0)	6.1
Protein (g/gCr)	1.43	TP (g/dl, 6.6–8.1)	11.7
Occult blood	(3+)	Alb (g/dl, 4.1–5.1)	2.6
Glucose	(−)	BUN (mg/dl, 8–20)	13.6
β_2_MG (μg/l, 5–253)	253	Cr (mg/dl, 0.65–1.07)	0.77
NAG (IU/l, 1.0–4.2)	22.1	eGFR (ml/min/1.73m^2^)	87.7
		UA (mg/dl, 3.7–7.8)	5.6
Complete blood count	–	Na (mEq/l, 138–145)	136
WBC (/μl, 3300–8600)	7600	K (mEq/l, 3.6–4.8)	4.1
RBC (× 10^4^/μl, 435–555)	413	Cl (mEq/l, 101–108)	103
Hb (g/dl, 13.7–16.8)	10	Ca (mg/dl, 8.8–10.1)	8.5
Plt (×10^4^/μl, 15.8–34.8)	37.1	IP (mg/dl, 2.7–4.6)	3.4
		AST (U/l, 13–30)	10
Serology	–	ALT (U/l, 10–42)	7
ANA	1:40	LDH (U/l, 124–222)	102
MPO-ANCA (U/ml, 0–8.9)	2.5	γGTP (U/l, 13–64)	13
Anti-SS-A (U/ml, 0–7.0)	1.3	CRP (mg/dl, 0–0.14)	7.65
Anti-SS-B (U/ml, 0–7.0)	2.1	IgG (mg/dl, 861–1747)	6940
SAA (μg/ml, 0–8)	1300	IgA (mg/dl, 93–393)	543
IL-6 (pg/ml, 0–4.0)	15.5	IgM (mg/dl, 33–183)	429
geniQ HHV8 (copy/ml, 0–2 × 10^2^)	undetectable	C3 (mg/dl, 73–138)	131
HIV antibody (S/CO, 0–1.00)	0.07	C4 (mg/dl, 11–31)	18.1
		CH50 (U/ml, 31.6–57.6)	49
		Ferritin (ng/ml, 50–200)	102.6
		sIL-2 R (U/ml, 122–496)	1276

*Alb* Albumin, *ALT* Alanine transaminase, *ANA* Antinuclear antibody, *Anti-SS-A* SSA antibodies, *Anti-SS-B* SSB antibodies, *AST* Asparate transaminase, *β2MG* β2-microglobulin, *BUN* Blood urea nitrogen, *C3* Complement 3, *C4* Complement 4, *Ca* Calcium, *CH50* 50% hemolytic complement activity, *Cl* Chloride, *Cr* Creatinine, *CRP* C-reactive protein, *eGFR* Estimated glomerular filtration rate, *γGTP* γ-glutamyltranspeptidase, *Hb* hemoglobin, *HbA1c* Hemoglobin A1c, *HCO*_*3*_^−^ Bicarbonate ion, *HHV8* human herpesvirus 8, *HIV* Human Immunodeficiency Virus, *IgA* Immunoglobulin A, *IgG* Immunoglobulin G, *IgM* Immunoglobulin M, *IL-6* Interleukin-6, *IP* Inorganic phosphate, *K* Kalium, *LDH* Lactate dehydrogenase, *MPO-ANCA* Myeloperoxidase antineutrophil cytoplasmic antibody, *Na* Natrium, *NAG* N-acetyl-β-D-glucosaminidase, *Plt* Platelets, *RBC* Red blood cells, *SAA* Serum amyloid A, *sIL-2* Soluble interleukin-2 receptor, *TP* Total protein, *UA* Uric acid, *WBC* White blood cells

**Fig. 1:**
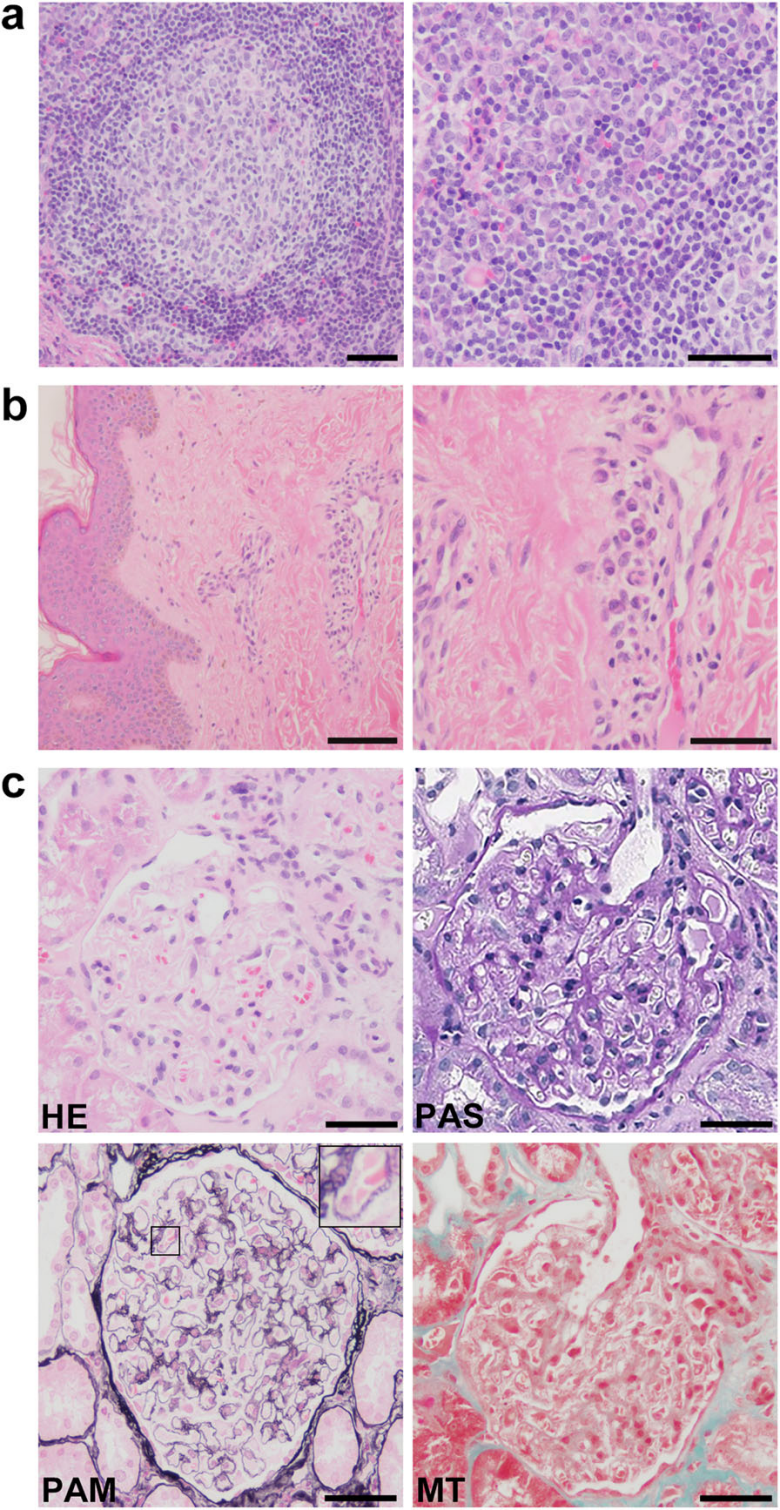
Light microscopy. **a** A submental lymph node biopsy showed diffuse interfollicular plasma cell infiltration (Hematoxylin and eosin staining [HE]). Left panel: Bar = 50 μm, Right panel: Bar = 50 μm. **b** Skin biopsy showed plasma cells in the perivascular area (HE). Left panel: Bar = 250 μm, Right panel: Bar = 100 μm. **c** Kidney biopsy findings. HE staining showed no sign of inflammatory cell infiltration in the glomeruli. Periodic acid Schiff (PAS) staining showed no signs of mesangial proliferation, crescents, or adhesion. Periodic acid methenamine silver (PAM) staining showed the appearance of bubbling (enlarged rectangle) in the glomerular basement membranes. Masson-Trichrome (MT) staining showed no sign of immune complex deposits in the glomeruli. Bars = 50 μm

An excisional submental lymph node biopsy showed diffuse interfollicular plasma cell infiltration (Fig. 1a). Immunohistochemistry revealed neither interfollicular plasmacytosis nor laterality of the κ or λ light chains. IgG4-positive cells were detected, but the IgG4/IgG ratio was < 0.1. A skin biopsy of a papule on the patient’s back showed plasma cells in the perivascular area (Fig. 1b). The patient was diagnosed with MCD, plasma cell variant.

**Fig. 2:**
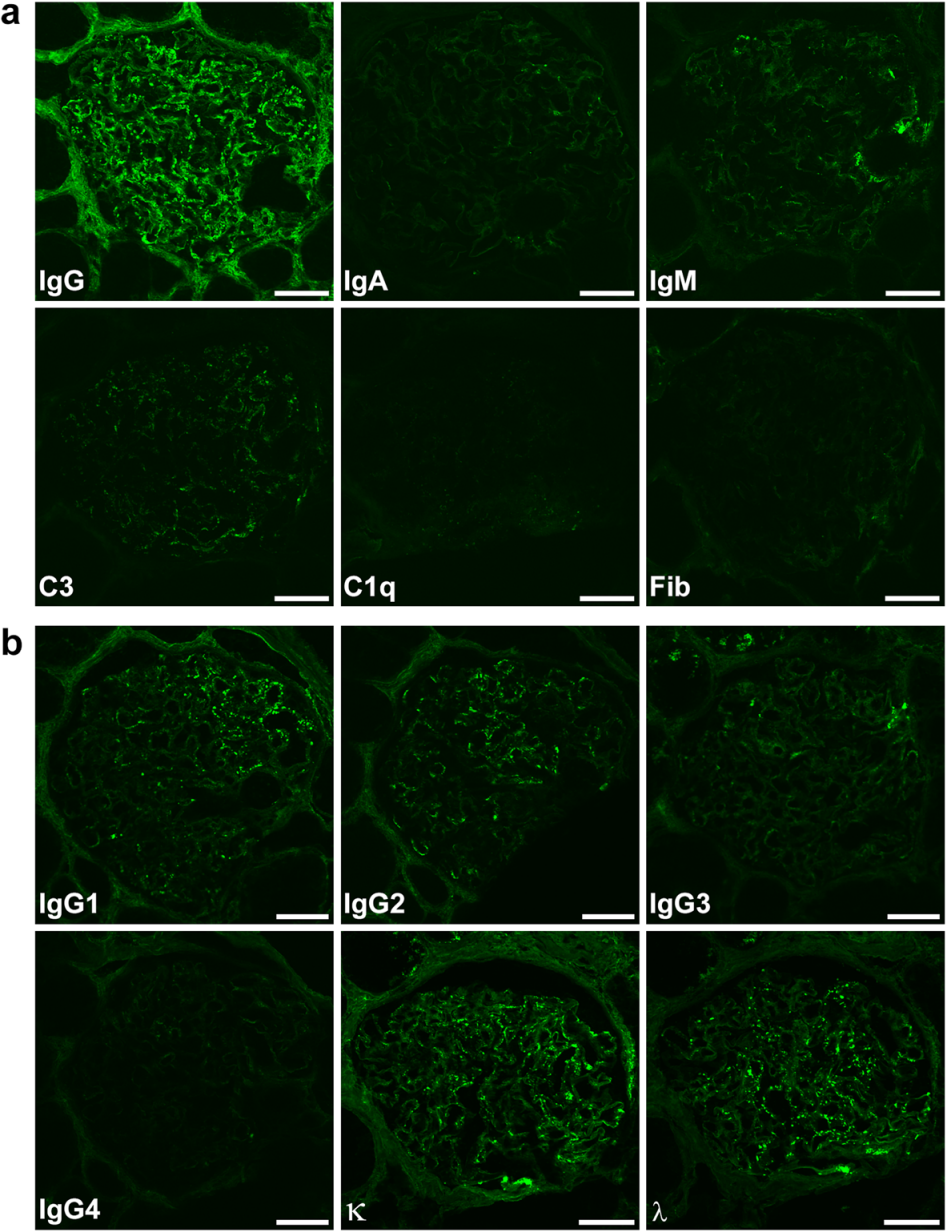
Immunofluorescence study. **a** Immunofluorescence showed strong focal granular staining for IgG and weak focal granular staining for C3 along the glomerular basement membranes. Immunofluorescence showed no signs of IgA, IgM, C1q or Fib. Bars = 50 μm. **b** IgG subclass staining was composed of predominantly IgG1 and IgG2, not IgG4. Bars = 50 μm. There was no major difference in staining between the κ and λ chains. Bars = 50 μm

**Fig. 3:**
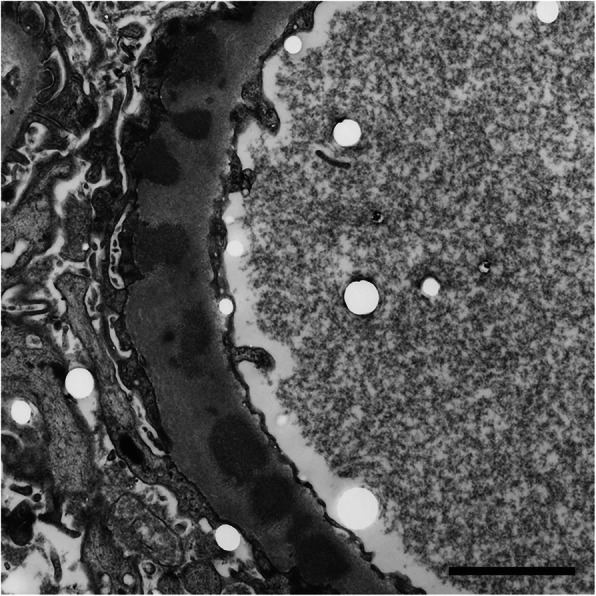
Electron microscopy. Electron dense deposits were observed within glomerular basement membranes. This was classified as Ehrenreich-Churg stage III. Bar = 2 μm

Kidney biopsy revealed 2 instances of global sclerosis in 19 glomeruli. Hematoxylin and eosin (HE) staining showed that there was no inflammatory cell infiltration in the glomeruli (Fig. 1c). Periodic acid Schiff (PAS) staining showed no signs of mesangial proliferation, crescents, or adhesion (Fig. 1c). Periodic acid methenamine silver (PAM) staining showed the appearance of bubbling in the glomerular basement membranes (Fig. 1c). Masson-Trichrome (MT) staining demonstrated the absence of immune complex deposits in the glomeruli (Fig. 1c). Immunofluorescence showed the focal granular deposition of IgG and C3 along the glomerular basement membranes (Fig. 2a). The IgG deposits were predominantly composed of IgG1 and IgG2, but not IgG4, suggesting secondary MN rather than primary MN (Fig. 2b). Anti-phospholipase A2 receptor antibodies were not measured. There was no big difference in staining between κ and λ chains (Fig. 2b). Direct fast scarlet staining was negative. Electron microscopy revealed electron dense deposits within and outside the glomerular basement membranes (Fig. 3). Based on these results, the patient was diagnosed with secondary MN.

**Fig. 4:**
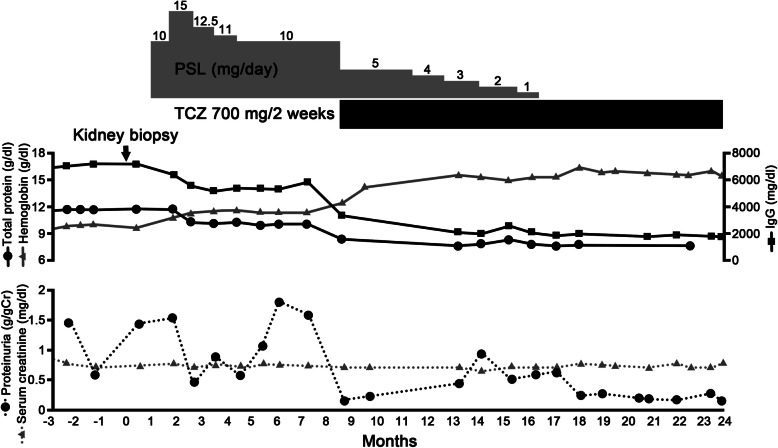
The clinical course

The patient was initially treated with prednisolone alone; however, his biochemical abnormalities did not improve. Thus, intravenous tocilizumab (700 mg every 2 weeks) was started. After treatment with tocilizumab, the patient’s C-reactive protein (CRP) elevation, anemia, and polyclonal gammopathy improved (Fig. 4). Furthermore, his urinary protein level declined from 1.58 g/gCr to 0.13 g/gCr. The prednisolone dose was gradually tapered, then discontinued. He has been stable without a recurrence of proteinuria for more than 6 months.

## Discussion and conclusions

We experienced a rare case of MN that was associated with MCD. The patient’s high zinc sulfate level was considered to be related to CD. In a kidney biopsy specimen, PAM staining revealed a bubbling appearance in the glomerular basement membranes and electron microscopy revealed electron dense deposits within and outside the glomerular basement membranes; these findings were compatible with a diagnosis of MN. Since the IgG depositions were predominantly composed of IgG1 and IgG2, the MN in the present case was thought to be secondary rather than primary.

Regarding renal complications of CD, 9–71% of CD patients are reported to have elevated creatinine or blood urea nitrogen [6]. Another report showed that 19 of 76 CD patients (25%) had renal involvement and that the most common etiology was TMA-like lesions [7]. Several studies reported that the renal histology of CD mainly included AA amyloidosis, TMA, and MPGN [4, 5]. CD-associated AA amyloidosis might be caused by the overproduction of IL-6 because IL-6 was reported to play a critical role in the synergistic induction of the human SAA gene [8]. With respect to CD-associated renal TMA, Mutneja et al. reported that the VEGF expression in podocytes was decreased despite there being a high level of VEGF in circulation [9]. Since the local reduction of VEGF within the kidney was reported to be sufficient to trigger the pathogenesis of TMA in adult mice [10], CD-associated renal TMA might be caused by the downregulation of VEGF in podocytes. Regarding CD-associated MPGN, the exact mechanism through which CD leads to MPGN remains unknown; however, MPGN related to CD might be secondary to chronic TMA [11].

We searched the PubMed database for relevant studies using the following search term: “castleman kidney” or “castleman membranous nephropathy” or “membranous nephropathy angiofollicular lymph node hyperplasia”. CD-associated MN was rare, in addition to the present case, only 7 cases were identified in the relevant English literature (Table 2) [7, 12–17]. Among the 8 reported cases (including the present case), seven patients were male; only one patient was female. All patients who were tested had high serum levels of VEGF, IL-6, and CRP. Six patients experienced proteinuria. The histopathological patterns of the 8 cases were as follows: hyaline-vascular (HV) type, *n* = 2; plasma-cell type, *n* = 3; mixed type, *n* = 1; and unknown type, *n* = 2 (Table 3). Six of the 8 cases had favorable renal outcomes. Only case 1 experienced acute kidney injury, resulting in chronic renal failure. The treatment options varied.

**Table 2:** Summary of reported cases of membranous nephropathy associated with Castleman’s disease

	Article	Age	Sex	TP (g/dl)	Alb (g/dl)	Cr (mg/dl)	VEGF (pg/ml)	IL-6 (pg/ml)	CRP (mg/dl)	IgG (mg/dl)	Proteinuria (g/day)
1	Weisenburger 1979	51	M	6.8	0.8	1.3	NA	NA	NA	2960	10
2	Ruggieri 1990	15	F	5	2.1	NA	NA	NA	NA	300	20
3	Komaba 2008 pat 1	46	M	11	2	0.65	NA	23.8	8.8	6070	1
4	Tazi 2012	45	M	NA	1.9	0.69	NA	NA	12	NA	7.2
5	Xu 2012 pat 15	56	M	NA	2.28	NA	NA	NA	14.8	NA	NA
6	Sun 2020 pat 12	44	M	NA	NA	NA	89.2	NA	NA	NA	NA
7	Furutera 2020 pat 1	58	M	8.7	2.1	1.2	NA	36	8.6	4156	4.4 (g/gCr)
8	**The present case**	43	M	11.7	2.6	0.71	467	15.5	7.65	6940	1.43 (g/gCr)

*Alb* Albumin, *Cr* Creatinine, *CRP* C-reactive protein, *F* Female, *IgG* Immunoglobulin G, *IL-6* Interleukin-6, *M* Man, *NA* Not available, *pat* Patient, *TP* Total protein, *VEGF* Vascular endothelial growth factor

**Table 3:** Summary of the outcomes of the reported cases

	Article	LN	Renal phenotype	Renal histological type	Treatment	Renal outcomes	Patient outcomes
1	Weisenburger 1979	NA	NS	MN	Symptomatic treatment	NA	NA
2	Ruggieri 1990	HV	NS	MN	Lymphadenectomy, CY, Indomethacin	Complete remission	Survive
3	Komaba 2008 pat 1	Mixed	Mild proteinuria	MN (IgG2 positive),Localized IN	PSL, TCZ	Proteinuria < 0.5 g/day	Survive
4	Tazi 2012	HV	NS	MN	PSL	Complete remission	Survive
5	Xu 2012 pat 15	PC	Hematuria, NS, ARF, RPGN	CG, MN	R-CHOP, HD, PE	Chronic renal failure	Survive
6	Sun 2020 pat 12	NA	NA	ATIN, MN	PSL, CY, Thalidomide, CyA	Normalized	Survive
7	Furutera 2020 pat 1	PC	NS	CG, MN	PSL, TCZ	Complete remission	Survive
8	**The present case**	PC	Mild proteinuria	MN	PSL, TCZ	Complete remission	Survive

*ATIN* Acute tuburointerstitial nephritis, *ARF* Acute renal failure, *CG* Crescentic glomerulonephritis, *CY* Cyclophosphamide, *CyA* Cyclosporin A, *HD* Hemodialysis, *HV* Hyaline-vascular type, *IN* Interstitial nephritis, *LN* Lymph node, *Mixed* Mixed type, *MN* Membranous nephropathy, *NA* Not available, *NS* Nephrotic syndrome, *pat* Patient, *PC* Plasma cell type, *PE* Plasma exchange, *PSL* Prednisolone, *R-CHOP* rituximab, cyclophosphamide, adriamycin, vincristine, and prednisolone, *RPGN* Rapidly progressive glomerulonephritis, *TCZ* Tocilizumab

Previous reports suggested the efficacy of tocilizumab in reducing proteinuria as a renal complication of CD [14, 17, 18]. There were three cases of MN caused by CD, including the present case, that were treated with tocilizumab (Table 3). Komaba et al. [14]. reported a case of mixed-type CD that was treated with tocilizumab while the case reported by Furutera et al. [17] and the present case were plasma cell-type CD. Although it has not been clarified why CD causes MN, the reduction of proteinuria in CD-associated MN by tocilizumab, which is an IL-6 receptor antagonist, suggests that CD-associated MN might be related to IL-6. While he has been stable without recurrence of proteinuria for more than 6 months, a longer follow-up period is needed, as the proteinuria in the present case previously became exacerbated 5 months after treatment with tocilizumab.

In conclusion, we experienced a case of CD-associated MN in which tocilizumab was effective for reducing proteinuria. Further reports should be accumulated to determine why patients with CD can present secondary MN as a renal complication, and why tocilizumab can reduce proteinuria in CD-associated MN.

## Data Availability

The datasets used and/or analysed during the current study are available from the corresponding author on reasonable request.

## Abbreviations

CD: Castleman’s disease (CD)
CRP: C-reactive protein
IL-6: Interleukin-6
MCD: Multicentric CD
MPGN: Membranoproliferative glomerulonephritis
TMA: Thrombotic microangiopathy
UCD: Unicentric CD
VEGF: Vascular endothelial growth factor
